# “Daring to deal with the difficult and unexpected” registered nurses’ confidential conversations with patients with palliative care needs: a qualitative interview study

**DOI:** 10.1186/s12904-023-01228-y

**Published:** 2023-07-31

**Authors:** Tove Stenman, Ulla Näppä, Ylva Rönngren, Christina Melin-Johansson

**Affiliations:** 1 Department of Health Sciences Nursing Science, Mid Sweden University, Östersund, Sweden; 2 Department of Health Sciences Nursing Science, Mid Sweden University, Östersund, Sweden; 3 Department of Nursing, Mid Sweden University, Östersund, S-831 25 Sweden

**Keywords:** Confidential conversations, Existential issues, Hospice, Nurse-patient-relationship, Palliative care, Qualitative research

## Abstract

**Background:**

In palliative care, registered nurses provide advanced nursing care to relieve patients’ symptoms and increase their quality of life based on physical, mental, social and existential dimensions. Conversations, often about existential issues, are an important part of nursing and can affect quality of life positively. Confidential conversations between patients and nurses occur naturally while other nursing activities are being performed. Despite their great importance for palliative care these are rarely described.

**Aim:**

To gain a deeper understanding of how nurses in palliative care experience and describe confidential conversations with patients.

**Method:**

Secondary analysis of data from 17 open-ended face-to-face interviews with registered nurses in palliative care was conducted. Qualitative content analysis using an inductive approach was used to gain a deeper understanding and analyse the latent content.

**Results:**

The confidential conversation was considered an important part of palliative care and is the nurse’s responsibility. This responsibility was described as complex and placed various demands on the nurses, both personal and professional. A prerequisite for the conversation was the interpersonal relationship. The conversation allowed the patient to process important matters not previously addressed or put into words. It had no predetermined content, was unplanned and entirely on the patient’s terms. For nurses the conversation could be experienced both as draining and a source of power and strength. The nurses also described safeguarding the patient through the conversation.

**Conclusion:**

Nurses’ confidential conversations with patients are essential in palliative care and must be highlighted more to increase the quality of palliative care. The confidential conversations often have an existential content and are challenging for the nurses. Therefore, nurses need time, knowledge, and supervision to increase their conversation skills.

## Background

Communication through conversation is essential for achieving the goals of palliative care, i.e., providing care and support to people with a limited life expectancy due to illness or old age. The communication and the relationships between registered nurses (RNs) and patients with palliative care needs (PPCN) are fundamental and have an ontological basis in the ethics of proximity [1, 2]. PPCNs have emphasized the importance of communication and that healthcare professionals should listen, and show compassion and respect [3]. Research shows that living with a life-threatening illness raises issues that PPCNs need to talk about [4, 5]. PPCNs´ psychosocial, spiritual, and existential symptoms and problems may be alleviated when they are given the opportunity to share their concerns with healthcare professionals [6]. Unfortunately, PPCN often lacks emotional and psychosocial support and these unmet needs are significantly associated with higher psychological stress [7–9]. Palliative care is person-centered, holistic, and preventive, with early identification, evaluation, and treatment of symptoms and problems that may be complex [6]. Several review studies have shown that the symptoms most commonly reported by PPCNs are pain, nausea, and fatigue, along with psychological and existential symptoms [7–11].

Increasing evidence is available regarding the importance of communication in palliative care, with RNs. This is nothing new, in the 19th century, communication was identified as an essential part of nursing [12]. In the International Council of Nurses’ (ICN) nursing definition states that RNs are responsible for meeting patients’ needs for care and support and highlights the importance of communication skills [13]. Different communication models are described in the literature. For example, *serious illness conversations* are described in one study as conversations between patients and their relatives and a physician about the patient’s goals, wishes, and priorities that will inform their future care [14]. The *prognostic conversation* is well described and is primarily the physician’s conversation with the patient and relatives giving them information regarding a transition point in the care and treatment that has been reached. The prognostic conversation delivers both a message and information. It may be followed by *an advanced care planning conversation*. This is also primarily a medical conversation in which physicians discuss treatment strategies and treatment limitations for future care with patients and relatives [15]. A *difficult conversation* is described as something essential and associated with life and is primarily about giving difficult or sad information [16, 17]. A *comfort conversation* is described by Konietzny and Anderson [18] as part of person-centred care where the patient’s holistic palliative care needs and symptoms are central. The conversation focuses on supporting feelings and needs, increasing well-being, or collaborating on the plan for future care. An *existential conversation* includes topics related to life, death, and relationships. This conversation is particularly important in the care of patients with life-threatening illnesses [19, 20]. However, a literature review and a qualitative meta-synthesis showed that spontaneous and naturally occurring conversations with RNs, and their content, are rarely described. These studies describe how this might be due to these conversations occurring while other types of care activities are being performed, making them both invisible and obvious [21, 22].

One survey described how RNs are aware of patients’ existential needs and have a key position to talk about existential issues with PPCNs. The RNs may receive first-hand information regarding the patients’ existential needs. But these conversations prompt the RNs to be responsive, present in the moment, honest, and open [23]. A systematic review shows that RNs find dealing with symptoms of a spiritual, existential, and psychosocial nature challenging due to lack of knowledge and time [3]. A previous intervention highlighted that training and reflection influence both the quality of palliative care and RNs confidence when communicating with patients about existential issues [24]. Two recent systematic reviews [25, 26] showed that educational interventions for RNs and nursing students concerning topics such as spiritual intelligence [26] and therapeutic conversation education [25] increased the RNs’ knowledge and self-confidence in conversations with existential content.

Available research shows that PPCNs have a strong need to talk about their situation and thoughts, but they experience their existential and psychosocial symptoms as unmet. Conversations that can be supportive and health-promoting are important in palliative care, by validating suffering and promoting health despite severe illness and impending death. RNs have a unique opportunity to provide support based on conversations in their close relationship with the patient. It is therefore of great importance that RNs create opportunities in their daily work for conversations, which should be held in confidence. Confidential conversations between PPCNs and RNs were first described in a master’s thesis written by the first author. The results sparked an interest in gaining a deeper understanding of the content of these conversations. Concepts like intimacy, closeness and trust are related to the concept of confidentiality and are by definition closely related. They are used to describe an exchange of current thoughts and feelings in a community between two individuals. Unfortunately, the importance of the RN’s confidential conversation is too little recognized. There is a lack of studies and knowledge about confidential conversations and interventions that can improve the RN’s competence in such conversations and raise the quality of palliative care. This study, therefore, aims to gain a deeper understanding of how RNs in palliative care experience and describe confidential conversations with PPCNs.

## Methods

A qualitative secondary analysis was carried out [27] of an existing data set derived from 17 face-to-face interviews with RNs in palliative care. The data was conducted for an unpublished master’s thesis written by the first author which described intimacy in palliative care. The interview questions included “how do you define intimacy”, “describe intimacy in palliative care in the nurse-patient-relationship” and “describe intimacy in palliative care in the patient-partner-relationship”. During the interviews follow-up questions were used to deepen the answers.

Focus in the secondary analysis was on confidential conversations that the nurses talked about in the interviews in the primary study, and was one important subcategory. New research questions were developed from the primary study which guided the analysis in the secondary analysis. Inductive content analysis was utilized in the secondary analysis of the data in order to gain a deeper understanding of such conversations [28, 29]. The secondary analysis process is described in Table 1.

**Table 1:** Secondary analysis process

	The primary study
	Conducted in 2019
Title	Intimacy in palliative care from a nursing perspective - A qualitative interview study
Aim	To describe nurses’ experience of intimacy in palliative care.
Design	A total of 17 open-ended face-to-face interviews with RNs in palliative care in the Northern Region of Sweden were analyzed using qualitative content analysis with an inductive approach [28, 29]
Interview questions	Describe intimacy in palliative care
Results	Category: Intimacy in the care relation Subcategories: - Bodily care - Confidential conversation - Home, care room, and possession as intimate Category: Intimacy in the close relationship with a partner Subcategories: - Conversation about sexuality - The changed body - Physical touch as symptom relief - Relatives as caregivers
Reference	Stenman, T. *Intimacy in palliative care from a nursing perspective. A qualitative interview study.* Master´s thesis. Ersta Sköndal Bräcke Högskola Stockhom: Sweden (Unpublished)
	**The secondary study**
	Conducted in 2022
New research questions developed from the primary study	How do RNs experience and describe confidential conversations? What can be raised in a confidential conversation? What conditions are described as necessary for a confidential conversation?
Aim	To gain a deeper understanding of how RNs in palliative care experience and describe confidential conversations with PPCNs
Secondary study	Secondary analysis (27) Qualitative content analysis with an inductive approach (28, 29)

### Settings and participants

The settings for the study were specialized palliative care units in municipal home care, palliative consulting teams, palliative inpatient care, and specialized teams that care for PPCNs in their home in the Northern Region of Sweden. The region is sparsely populated and includes four counties with approximately 900,000 inhabitants in total [30]. In this part of Sweden, specialized palliative care is provided in an inpatient ward or in the patient’s own home in both rural and urban areas. Within the catchment area for this study there were two hospices, one hospital ward with round-the-clock care, and five home care teams that provided specialized palliative care. The care of PPCNs in their own home was carried out in close cooperation with municipal health care. The teams were organized differently in different parts of the region. They comprised both specialist-trained and general nurses, as well as doctors, with some teams also including social workers, occupational therapists, and physiotherapists [31].

Purposeful sampling was used. Six administrative managers of specialized units were sent an information letter and asked to forward the request for participation in the study to the RNs at the unit. The inclusion criterion was that they cared for PPCNs at the end of life who were connected to a specialized palliative unit. Seventeen RNs participated. See Table 2 describing the demographic profile of the sample.

**Table 2:** Demographic profile of sample

N = 17	N (%)
Gender	
Female/male/other	12 (70%) / 5 (30%) / 0
Age	29–63 years
Mean	49 years
Years working as a nurse	2.5–42 years
Mean	21 years
Years working in palliative care	0.75–20 years
Mean	11 years
Care settings	
Inpatient ward/ Home healthcare	6 (35%) / 11 (65%)
Education	
Graduate without further education/Graduate with further education	5 (30%) / 12 (70%)

### Data collection

Data were collected using face-to-face interviews with the support of a semi-structured interview guide containing open and follow-up questions. This was intended to encourage the participants to reflect and to deepen their thoughts, and to ensure that the information collected fulfilled the aim of the study [32]. Questions included for example “describe intimacy in palliative care in the nurse-patient-relationship” and “describe intimacy in palliative care in the patient-partner-relationship”. When the RNs talked about intimacy in the nurse-patient relationship, they gave a rich description of an intimate, confidential conversation that was part of an intimate care relationship between themselves and the PPCNs.

The RNs were invited to choose the place and time for the interviews, all of which were carried out at the RNs’ workplace. The interviews began with a brief presentation of the author and verbal information about the study, the purpose of the study, and that participation was voluntary. Everyone was invited to answer demographic questions; the results are presented in Table 2. The interviews lasted between 23 and 69 min (median 43 min) and were audio-recorded digitally. Once all respondents had been interviewed, the interviews were transcribed verbatim and stored on a password-protected server at Mid Sweden University.

### The secondary data analysis

The purpose of the secondary analysis was to address new research questions inspired by the analysis of the results in the master’s thesis described above [27, 33]. The content of the interviews was considered to be rich and to contain more information that could subsequently be analysed in the present study to gain a deeper understanding of the confidential conversations described [34]. Heaton’s [27] recommendations were followed during the planning for the secondary analysis to ensure the integrity of the data collected and an honest intention. This involved evaluating whether the data from the primary study (the master’s thesis) and the secondary research questions were compatible. The analysis has been transparent at all stages, which Heaton believes is central when using secondary analysis. The purpose, data collection, and analysis of the thesis are described below.

### Content analysis

Inductive content analysis was utilized with a focus on finding patterns (28, 29). Descriptions of the content of and conditions for confidential conversations were of particular interest. The interviews were read by all authors in the present study to gain an overall picture and a broad understanding. During the inductive analysis, new perspectives emerged which differed from the analysis in the thesis. Meaning units that corresponded to the study’s aim were identified and marked. The meaning units were condensed, abstracted and sorted into codes. All codes were compared and then divided into themes; the themes were compared with the interviews to ensure that the interpretation was consistent with the text as a whole. The themes were further analysed to describe the underlying meaning (28, 29) and an overarching theme was analysed which connected to the latent content.

### Ethical considerations

Ethical approval for the study was obtained from the Ethical Review Authority in Sweden (Dnr 2021–04066). Ethical considerations followed the research ethical rules presented in the Declaration of Helsinki [35].

All the RNs who participated received written and verbal information about the study. The administrative managers signed an agreement allowing the RNs to take part in the study. All RNs signed informed consent and received information that participation was voluntarily and that they could terminate participation without giving a reason. The unit manager would be contacted if the RNs wanted support due to strong emotions being aroused during the interview. Once all the interviews had been completed, they were transcribed verbatim and stored according to good research practice and ethical principles [35].

The participants could not be contacted for permission to analyze data a second time as no information about e-mail or telephone numbers had been stored. -Therefore, no additional informal content about participants was collected before the secondary analysis.

## Results

The overarching theme from the analysis covers the processing of fundamental issues about life and death in confidential conversations between RNs and PPCNs during palliative care. The RNs described that the content addressed in confidential conversations was complex, including concepts such as intimacy, unforeseeable, emotional, challenging, safeguarding, and performed within a limited time frame. The overarching theme, themes, and subthemes are presented separately below, with all being interconnected. (see Table 3). Each theme is illustrated with quotes from participants.

**Table 3:** The overarching theme, themes, and subthemes related to the RNs´descriptions of confidential conversations

Overarching theme:	
**The confidential conversation - To gain trust when sharing urgent issues about life and death**	
Themes:	Sub themes:
Remaining in the unpredictable	Being certain in the uncertain content
	Reflecting the emotional
Managing the complex responsibility	Being brave and careful
	Listening but not solving
Safeguarding the PPCN through the conversation	Protecting the vulnerable patient
	Preserving integrity and hopes
	Confirming thoughts and feelings

### The confidential conversation – gaining trust when sharing urgent issues about life and death

The RNs tried to gain trust in several ways when PPCNs narrated their life stories and the confidential conversation was considered as a source of healing for the PPCNs. The RNs aimed to promote a good death, a dignified farewell to life, and ensure that the PPCNs felt valued to the end. They strove to accommodate PPCN’s wants and needs and to find time for confidential talks.

### Remaining in the unpredictable

The confidential conversation was described as unpredictable both in content and moment, despite that they had to stay and be present. The RNs described how they had to be certain in an uncertain situation and had to control their emotions even if they were emotionally affected.

#### Being certain in the uncertain content

Since the conversations were initiated by PPCNs, often in the context of RNs’ nursing activities, it could lead to RNs feeling unprepared and confused. But they knew that they should remain despite the uncertainty because they knew that the conversation was important. RN no 110 described it like this:

“...yes, it is completely based on the patient’s wishes that they...talk about what they want to talk about, that they receive help and support to talk about it. It is often that they do not do or dare to do it themselves... or at least that they get to start with someone who supports...“ (RN no 110)

The content of the conversation differed both from patient to patient and from occasion to occasion. Questions were often raised that could not be predicted or prepared for. The unpredictable content created a sense of uncertainty. The RNs described that they needed to deal with the situation then and there by finding inner certainty.

The presence of death and dying influenced the conversation. The content was characterized by the PPCN’s life experiences and their need to process difficult life situations which they had not processed earlier in life. Spiritual and existential issues, such as life and death, faith and doubt, and deeply personal thoughts, were common subjects. Also, thoughts about the future, such as impending death, dreams, and hopes, were common subjects. The RNs described how the uncertain content could be perceived as sad and burdensome, but at the same time inspired and curious.

Sometimes strong mixed emotions such as crying, anger, frustration, or laughter and joy were aroused during the conversations. However, the RNs never knew in advance how the PPCN was going to react, which could lead to a feeling of uncertainty.

#### Reflecting the emotional

The RNs claimed that the conversations could stimulate a variety of emotions. The emotions could surprise and were sometimes perceived as frightening. But above all, RNs emphasized that the conversation was an intimate meeting which was experienced as a gift, they could feel honored as one RN described:

“You get the feeling that this person hasn’t talked to many people about this... So it becomes... well... it becomes a trust and it’s like time stands still somehow. You forget a bit of time and space when you enter into such a conversation... I think you could say that there is some kind of intimate feeling then.” (RN no 105)

The RNs listened with respect and curiosity, when PPCNs narrated their life stories and shared their life wisdom. A common theme was that the RNs considered these conversations to be an existential challenge that could help them mature both personally and professionally. Reflecting on their own thoughts about life and death made them stronger as a person. They dared to address these issues, drawing strength from and being open in the conversations. One RN described that:

“...sometimes I have a lot of demanding conversations... then I’m completely exhausted when I get home. But I can also get renewed energy if the conversation was a nice moment when the patient and I had established a good connection...“ (RN no 101)

Finding different strategies to handle the conversations was a necessity, but also helpful. At the same time it was challenging and frustrating, and the RNs described that they tried to put their emotions aside, carry on, empathize with the PPCN, and not have any preconceived ideas.

### Managing the complex responsibility

The RNs stated that the confidential conversation was their responsibility. To deal with, they had to be brave but careful, actively listen and not try to solve PPCN’s problems.

#### Being brave but careful

The RNs described that the conversation required courage, but at the same time being careful. It took courage to dare to lead the conversation further by asking uncomfortable questions, knowing the importance of the conversation and the limited time left in PPCN’s life.

“In the conversation... you may have to feel the boundaries a little and push a little to see where... how strong that integrity is. ... without offending them. So it’s very delicate if you don’t have a lot of time, if things go downhill fast and they die quite quickly like that.” (RN no 104).

At the same time, they wanted to safeguard PPCN and their relationship. It was described as difficult to know how far the conversation could be challenged without affecting the relationship negatively. One RN described it as follows:

“it’s a lot about relationships and daring, daring to deal with the difficult” (RN no 107).

The relationship was brought up as an opportunity and sometimes a prerequisite for the conversation, but too close a relationship could be an obstacle. Using personal experiences and alternating between being professional and private was described as effective, but required courage and caution to protect the boundaries between the two. The RNs interpreted different signals and used their own intuition as support. Intuition was described as implicit, an art that could not be explained or taught but that grew with experience, helping them balance between courage and caution.

#### Listening but not solving

Two different aspects of taking responsibility in confidential conversation was to listen actively but not solve the PPCN´s problems. Part of listening actively was being present and seeing the individual in front of them which required the RNs to be quiet.

“... there is a reason why you have been given two eyes and two ears and one mouth. To look... to see who you have in front of you... listen to what they say… and realize that my words are not important right now.” (RN no 107)

Part of not solving the PPCNs’ problems was not to solve conflicts described by PPCNs even if it gave the RNs feelings of guilt. In these conversations, RNs felt their words were redundant, reinforcing the belief not to resolve or provide answers. As one RN put it:

“... it has turned out that they have a bad conscience because they haven’t been around or there are conflicts that... and we can’t, we can’t solve them... we can be there, but we cannot resolve any conflicts.” (RN no 115)

Not coming up with solutions or answers could be challenging at times and it was important for the RN to understand which conversations and questions could and could not be resolved. It was difficult to know how much to talk and what to answer. However, there were some issues that had to be addressed, such as fear and if the RN provided appropriate and accurate information in a professional way, the PPCN felt at ease. Upholding a professional approach meant daring to remain silent and not respond or give advice. Having clinical and life experience, and feeling secure facilitated these conversations.

### Safeguarding the PPCN through the conversation

The RNs described how they tried to safeguard the PPCNs through the conversation by protecting them, preserving their integrity and hopes, and.

confirming their thoughts and feelings.

#### Protecting the vulnerable patient

RNs perceived PPCNs to be frail, fragile, vulnerable, lonely, unprotected and vulnerable in their situation. One RN stated:

... the people we care for are so fragile. Most of them are in crisis and are sad and... they are very exposed and don’t have their usual authority and protection, they are sad and a little broken, empty and tired (RN no 103).

This fragility and vulnerability prompted an instinct to protect the PPCN in various ways. Concepts such as autonomy and self-determination were important components of both conversation and relationship, something that was important to guard. A part of safeguarding the PPCNs was to protect them from strong emotions and difficult conversations, therefore some of the RNs avoided having confidential conversations at particular times, such as late evening or at night.

Humor and laughter were used as sources of positive energy to protect PPCN. RNs described laughter as a caring act that was restorative and soothing. They reflected on whether it was ethically and morally right to laugh and joke with PPCN. One RN shared his thoughts

“... is it right to have so much fun? Is it unethical?... should you be serious and talk about death instead...” (RN no 111)..

#### Preserving integrity and hopes

Preserving the PPCN’s integrity throughout the conversation involved being cautious and receptive. The RNs described how it was their responsibility to preserve the PPCNs´ integrity, especially if the PPCNs themselves lacked the ability to do so. The RNs occasionally needed to challenge the integrity when it was described as a barrier and an obstacle in the confidential conversation.

The deteriorating body was often the focus of palliative care and the RNs tried to focus on what was still intact. However, RNs felt that in order to preserve health, it was important to minimize the risk of upsetting the PPCN emotionally. By talking about everyday topics, such as interests, memories, and personal belongings, and about what was still intact in the body, RNs tried to preserve the healthy. Conversations about health illuminated the RN’s desire to preserve hope by helping PPCNs find new vicarious hopes. The RNs could help and support the PPCN to find hope through conversations about the future, desires, hopes, fears, and concerns.

“It’s cancer and ascites and nausea and... it’s so easy to slip into that, to talk about symptoms. When you get to talk about something that is… well… intimacy or their relationship, this healthy. Something that can still be influenced for the better even, a little bit hopeful...” (RN no 103)

#### Confirming thoughts and feelings

The RNs described that it was natural to confirm the PPCN’s need for conversations, concerning emotions and thoughts about their self-image. When PPCNs dared to expose themselves and show their “ugly me”, showing a side of themselves that no one had previously seen.; was understood as an expression of trust. The RNs then confirmed the PPCN even though it was difficult for them to talk about their deteriorating body. In such conversations, it was important to be honest and confirm the PPCN´s feelings, which in itself was challenging, as one RN said

“...but I think if you have difficulty showing your own body and you can’t accept it because of illness and such...then you have to try to accept it and help maintain that confidence, but at the same time try to help too, to give support in that...it’s difficult... Yes, how do you support a deteriorating body...” (RN no 109).

Another part of confirming the PPCNs’ was by talking about the healthy, such as the PPCN’s needs, desires, and dreams of the present and future. The RNs often intuitively felt that the PPCN wanted to talk and that the conversation could be healing. If the RNs asked questions or using something personal to open up for further conversation.

## Discussion

The aim of the study was to gain a deeper understanding of how RNs in palliative care experienced and described confidential conversations. The main findings elucidate the importance of creating an interpersonal relationship, regardless of time and occasion. The RNs described how the conversations were unpredictable, and often took place during the performance of other caring activities. They also experienced that the confident conversation could be a way to safeguard the PPCN.

### The importance of an interpersonal relationship

The confidential conversations are about gaining trust enabling the PPCN to share existential issues before it is too late. To gain trust the RN create an interpersonal relationship with the PPCN. The relationship with the patient and their relatives is the very hub of palliative nursing, as described in Davies and Oberles’ theory of supportive palliative care [1, 2]. Their model presents different dimensions that have a great impact on supportive care which can be secured by creating and maintaining relationships (*contact*) with the patient and relatives. Tarbi et al. [19] state that the care relationship is a source of connection and togetherness in palliative care that influences the conversations. It is central to RNs ability to meet the patient’s emotional needs [36], and are crucial for the interaction between RNs and PPCNs [4, 5]. According to Moran et al. [21] the PPCN can talk confidently about difficult topics in a trustful relationship. In these arguments, the relationship and the conversations have a great impact on each other, and cannot be separated.

### Confidential conversations promote existential health and a healing

The confidential conversation is an important element of existential support. Moran et al. [21] state that during various nursing activities, for example, assistance with personal hygiene or wound dressings, RNs also perform several nursing actions that are rarely highlighted. RNs talk, observe, affirm, identify needs, and laugh together with the patients in order to create close relationships. In these naturally occurring conversations, existential questions arise to varying degrees [21] which the RNs in the present study also experienced. This everyday conversation, or small talk, can be a source of increased understanding and knowledge about the patient’s fears and may improve the patient´s quality of life [37]. In palliative care, RNs have a unique possibility to provide important opportunities for patients to talk about existential and spiritual issues [38]. Sekse et al. [22], confirm that and states that RNs provide the PPCN with existential and spiritual support through conversations. PPCNs receive help in managing their life situation, including illness, losses, and dependence on help and support, as well as increased quality of life and a sense of independence and dignity [22]. This has also been identified by Meier et al. [39] as important characteristics that affect the experiences of a good death.

### Confidential conversations are challenging and empowering

The confidential conversation offers enormous potential to meet the needs of PPCNs from a holistic perspective and is an important piece of the puzzle in palliative care. The RNs in our study described that death and dying were at the forefront of the confidential conversation since PPCNs had limited time left in their life. They experienced that the conversations often included existential issues, and RNs described that it could affect them emotionally. Both Gillman et el [36] and Powell et al. [40] argue that this is inevitable because the RNs are exposed to situations and events that arouse strong emotions. In our study, the RNs felt that to manage these complex responsibilities they need courage, knowledge, experience and personal skills. Gillman et al. [36] confirm this and believe that an individual’s ability to handle strong emotions that arise in difficult situations is dynamic and influenced by personal, environmental and contextual factors. Davies and Oberle [1, 2] refer to the concept of *attitudes*, arguing that attitudes are pervasive and concerned with approach and experience. Attitudes affect the entire relationship and are described as one of the dimensions of the theory of supportive palliative care [1, 2]. Experience and knowledge, support from the team, and time is necessary to dare to raise existential questions [36, 41]. Tornöe et al. [42] add courage since feelings of fear and uncertainty can arise. They argue that learning to be quiet, to listen, and be present in the moment increases the RN’s courage to dare to stay in the conversation.

RNs are aware that the effort they put into creating relationships also becomes a source of strength for themselves [36], which is confirmed in this study. The RNs described a feeling of being honored and chosen, which could strengthen them in their role. Even the conversations were described as a source of new energy for the RNs while safeguarding PPCN. According to Davies and Oberle’s [1, 2] model safeguarding involves helping the patient to find meaning by managing their illness or situation (*finding meaning*), performing nursing actions that the patient or relatives want help with (*empowering*), and using the appropriate resources for the physical care (*doing for*). The value and integrity of the patient can then be preserved and safeguarded. Safeguarding one’s value and wholeness (*preserving own integrity*) is also central in Davies and Oberle’s model and is the foundation of nursing [1, 2]. The present study shows that by the RNs preserving their own integrity and protecting their own boundaries, an opportunity is created to be fully present in the encounter with the patient, which is also confirmed by Moran et al. [21].

### Confidential conversations and holistic health of PPCN

The RNs described that by confirming, preserving, and protecting in the conversation, the PPCN could feel strengthened and able to share their emotional burden. Wang et al. [43] describe how a lack of conversation with RNs can lead to the patient feeling neglected and unsure about their value and sense of belonging. The patient´s various feelings should instead be confirmed as being normal [20, 37] in order to preserve their values, desires and life situation [21]. The RNs were also able to confirm and preserve the PPCNs´ integrity by allowing them to talk about the future, even though the time remaining was limited. This was perceived as empowering and could lead to maintaining dignity and wholeness, also confirmed by Elina et al. [44]. Siegle et al. [45] believe that patients living with life-threatening diseases have different ways of coping and managing information, something that can change over time and during the course of the disease. It is, therefore, important that healthcare professionals are flexible and can adjust the conversations and the information given as appropriate [45]. Michael et al. [46] also emphasize a patient’s ability to adapt and cope with illness and impaired function. Adaptation and coping can be facilitated if the patient is treated with care, goodwill and trust. In conversations with patients, existential questions need a place where the patient’s values and life goals can be used as a starting point for optimizing care [46].

### Methodological considerations

A secondary analysis of data entails some methodological considerations [27], but the interpretative approach to the analysis gave an deeper understanding on other aspect that was only partially addressed in the primary. The data that was used had substantial and rich content, and efforts were made to use the primary data to the maximum and find the latent content. When data is to be reused in a secondary analysis it is important to keep the ethics of informed consent in mind [47].

Conversations with PPCNs about life and death are sometimes perceived as abstract and complex. The data that was used had substantial and rich content, and efforts were made to use the primary data to the maximum and find the latent content. When data is to be reused in a secondary analysis it is important to keep the ethics of informed consent in mind [47].

The present study was based on interviews with nurses working in palliative care in order to ensure quality and trustworthiness, as described by Graneheim et al. [48]. During the study, the pre-understanding for as open-minded an approach as possible was discussed. All members of the research group are RNs with experience in healthcare where conversations are common. In order to ensure credibility and authenticity in the analysis [48], categories and themes were created with different levels of abstraction, which were strengthened with quotations. For accuracy in the analysis, the first author coded the preliminary categories which were then discussed with the research team. Group comments and understanding of themes were completed as part of the reflexivity process.

### Strengths and limitations

A strength of this study was the rich descriptions in the data set of conversations, which is to be discussed before the secondary analysis according to Heaton [27]. The research group assessed that the existing data were sufficient and could answer the new research questions. It may, however, be a limitation that the data collection took place based on research questions and the purpose of the primary study. Although specific in-depth questions about the confidential conversation were not used, the chosen method of analysis gave the opportunity for new, interpretive analyses.

Heaton [27] discusses the limitations of secondary analysis from an ethical perspective and with a particular focus on consent. In the present study, informed consent was collected during the data collection in the primary study. For the secondary analysis no additional consent could be obtained. The decision was justified by the fact that the research questions in the secondary analysis were close to the original research questions.

### Conclusions and clinical implications

Confidential conversations between PPCNs and RNs are crucial. They can make a huge difference in supporting the PPCNs in a good death and need to be highlighted more in order to increase the quality of palliative care. It is a conversation that is implicit and one that is not documented, and its importance is rarely emphasized. The context affects the content of the conversation since PPCNs often need to talk about existential issues. However, it is precisely such needs that PPCNs describe as being among the most unmet. Limited remaining time in life characterizes the confidential conversation when trust arises and the patient is given the opportunity to talk about urgent issues. For RNs the confidential conversation is a challenging yet important part of the puzzle in palliative care. However, for the conversation to take place, RNs need knowledge, training, supervision, support, and sufficient time. Under those conditions, it is possible to exploit the full potential of confidential conversations. Further research is needed to understand how PPCNs experience confidential conversations in terms of existential support. Practice and education in conversations are important and should be part of the curriculum for students in undergraduate and specialist nurse training.

## Data Availability

The datasets used and/or analyzed during the current study are available from the corresponding author under the prerequisite that no sensitive, personal or confidential data is revealed.

## Abbreviations

RN: Registered nurse
PPCN: patient with palliative care needs
